# Characterization of polymorphic microsatellite loci in the Antarctic krill *Euphausia superba*

**DOI:** 10.1186/1756-0500-7-73

**Published:** 2014-02-03

**Authors:** Rui Candeias, Sara Teixeira, Carlos M Duarte, Gareth A Pearson

**Affiliations:** 1 Centre of Marine Sciences, CCMAR-CIMAR, University of the Algarve, Gambelas Campus, 8005-139 Faro, Portugal; 2 Instituto Mediterraneo de Estudios Avanzados, IMEDEA (CSIC-UIB), Miquel Marqués 21, 07190 Esporles, Baleares, Spain; 3 The UWA Ocean Institute and School of Plant Biology, The University of Western Australia, 35 Stirling Highway, 6009 Crawley, Australia

**Keywords:** Antarctic krill, Genetic diversity, *Euphausia superba*, Pelagic invertebrate, Microsatellites

## Abstract

**Background:**

The Antarctic krill, *Euphausia superba* is a pelagic crustacean, abundant in high-density swarms (10 000 – 30 000 ind/m^2^) with a circumpolar distribution and a key role in the food web of the Southern Ocean. Only three EST derived microsatellite markers have been used in previous genetic studies, hence we developed additional highly polymorphic microsatellite markers to allow robust studies of the genetic variability and population differentiation within this species.

**Findings:**

The microsatellite markers described here were obtained through an enriched genomic library, followed by 454 pyrosequencing. A total of 10 microsatellite markers were tested in 32 individuals from the Antarctic Peninsula. One of the tested loci was fixed for one allele while the other was variable. Of the remaining nine markers, seven showed no departure from Hardy-Weinberg equilibrium. The mean number of alleles was 14.9.

**Conclusions:**

These markers open perspectives for population genetic studies of this species to unravel genetic structure, dispersal and population biology, vital information for future conservation.

## Findings

The Southern Ocean, comprising around 20% of the oceanic surface of the Earth, is considered to play a crucial role in the regulation of our planet’s climate [1] and is extremely vulnerable to global climatic change. One of Southern Ocean’s most important species is the Antarctic krill (*Euphausia superba*, Dana 1852), a shrimp-like crustacean that plays a central role in this ecosystem, being both a grazer of phytoplankton, bacteria and micro-zooplankton, and prey for vertebrates such as fish, seabirds, seals, penguins and whales [2,3]. It has a circumpolar distribution and is found in high-density swarms of 10,000 to 30,000 ind/m^2^. Krill is an important economic resource with estimated catches of 125,000 to 150,000 tons/year (CCAMLR, Commission for the Conservation of Antarctic Marine Living Resources). There is a serious concern about the combined effects of climate change, ocean acidification and fishing pressure on krill, and consequently on the vulnerability of the communities that depend on them [4].

For conservation and management purposes, knowledge of the genetic diversity and population differentiation of the target species is fundamental. To date some studies have addressed population differentiation in *E. superba*, using allozymes, mitochondrial DNA and EST-linked microsatellite markers [5-11]. However, the availability of microsatellite markers is limited and their development has proven difficult, as reported by other authors [10]. This could possibly be linked to the low GC content of the krill genome (32%), and its susceptibility to damage via UV-B radiation [11]. The main findings of the genetic studies performed to date suggest genetic homogeneity at large geographical scales. However several exceptions have been found, such as evidence of small-scale genetic heterogeneity found using mitochondrial SNP’s [12], and weak but significant genetic differentiation between populations [8]. Some authors suggest that temporal variability might explain the genetic differentiation found [10].

Here we report the development and characterization of polymorphic microsatellite loci for *Euphausia superba.* These markers will be useful for population genetic studies of this species, with the potential to provide fundamental information for conservation studies and management.

Whole genomic DNA was isolated using the CTAB method [13] from three individuals of *E. superba,* from muscle tissue kept at -80°C. To isolate the microsatellite sequences, a combination of an SSR-enrichment protocol with 454 pyrosequencing was performed by a commercial company (Ecogenics GmbH, Zürich, Switzerland). One CT/GT enriched library was generated, the insert size of the libraries was 500-800 bp and the length of 454 reads was 233 bp. A total of 38396 sequences were obtained, of these 1286 had microsatellite repeats. A total of 210 primer pairs were designed using primer3 core [14] of which 48 primer pairs were delivered by the service provider following amplification tests on three individuals. We tested all primers for polymorphism using a panel of 7 individuals from the Antarctic Peninsula. An M13-tail (TGTAAAACGACGGCCAGT) was added at the 5’ end of all forward primers to enable fluorescent-dye labelling [15]. Of all the primer pairs tested, we were able to optimize 10 polymorphic markers (Table 1).

**Table 1:** **Characterization of the polymorphic microsatellite loci identified in *Euphausia superba***

**Locus**	**Repeat motif**	**Primer sequence (5′-3′)**	**T**_**a**_**(°C)**	**Allele size range (bp)**	***A***	***H***_***O***_	***H***_***E***_	***F***_**IS**_
Esup1	(TGTA)_10_	F: TGTTTGGGTACTCACGGTCG	58	141-188	12	0.90	0.89	−0.006
		R: ACGTACATCCTCAGACAGAC						
Esup2	(TAG)_7_	F: ACCATAACCTCACTGACACC	57	103-125	5	0.36	0.37	0.053
		R: ATGGTAAGGTTGCACTGGAC						
Esup3	(GT)_11_	F: CGCATGATTGCATCGCAAAG	56	183-205	12	0.84	0.86	0.029
		R: AGGCACTCTCTGTCTCACTC						
Esup4	(AC)_14_	F: AATTTGAGAAAGCATAATACACTGAC	52	185-221	15	0.84	0.89	0.057
		R: TGTGTTTGTTGTGCATGTTTGAG						
Esup5	(GTA)_7_	F: CCAGTACCAACACTAGCACC	50	147-165	7	0.84	0.74	−0.14
		R: GCTGCACTCATTCCATCCAC						
Esup6	(TTG)_7_	F: CTATGGCGCCCACAAATTCC	50	215-279	17	0.77	0.92	**0.157**
		R: ACAACAGCAAGAGCATCCAC						
Esup7	(AG)_13_	F:TGCATAGATGTACAAAGAGATAGC	55	110-138	14	0.87	0.90	0.038
		R: ATATCCGCATCGGCAAACAC						
Esup8	(TG)_16_	F: GCATCAGGCTATGTTGAGGG	58	117-143	14	0.74	0.81	0.086
		R: TGAATCACATGCCAATACACAC						
Esup9	(TG)_12_	F:TTCTGGTGCCTATGAAGGGG	58	180-270	28	0.81	0.96	**0.167**
		R: TGCTCAATGAAATATGCAATAAAGAC						
Esup10	(TATC)_9_	F: AGCGCTATAATATCAAAAATACAACC	54	216-282	17	0.87	0.93	0.066
		R: AGAAAGTCCCTTTTTGCACG						

The following details are reported: name, motif, primer sequence and annealing temperature (Ta°C). Also descriptive statistics are presented, number of alleles *A*, observed (*H*_*O*_*)* and expected (*H*_E_) heterozygosities and heterozygote deficiency (*F*_IS_) according to Hardy-Weinberg equilibrium. Bold numbers indicate significant values at 0.1% level.

Amplification reactions in 10 μL contained 10 ng of genomic DNA, 1x Qiagen HotStart *Taq* buffer, 200 μM of dNTP’s, 0.04 μM of forward primer, 0.16 μM of reverse primer and fluorescently-labeled M13 primer, and 0.5 U of HotStart *Taq* polymerase (Qiagen). PCRs were conducted in a Perkin-Elmer GeneAmp7200 (Waltham, MA, USA) with the following program: 15 min at 95°C; 30 cycles composed of 30 s denaturation at 95°C, 45 s at the annealing temperature (Table 1) and 45 s elongation at 72°C, followed by an additional 8 cycles composed of 30 s of denaturation at 95°C, 45 s at 53°C and 45 s elongation at 72°C. A final 30 min elongation step at 72°C was performed. PCR products were amplified with M13 primers end-labelled with different fluorescent dyes, FAM, ATT550 or HEX to allow multiplexing. Fragments were separated on an ABI3130XL automated sequencer (Applied Biosystems, Foster City, CA, USA) with Rox350 size standard. Alleles were scored using Peak Scanner 1.0 (Applied Biosystems).

The variability of the markers was tested on 32 individuals sampled around the Antarctic Peninsula (between 69.1489°S; 73.526°W and 61.740°S; 53.7692°W). The number of alleles per locus (*A*), the observed (*H*_O_) and expected (*H*_E_) heterozygosities (Table 1) and the heterozygote deficiency (*F*_IS_) were calculated using the software GENETIX 4.05 [16]. The majority of the optimized markers (9) were highly polymorphic while in locus Esup5 one allele (147) is present in all individuals while there is variation in the other allele sizes, potentially limiting its usefulness for population genetic studies. The number of alleles found for the 9 loci ranged from 5 (Esup2) to 28 (Esup9); *H*_O_ ranged from 0.36 to 0.90 and *H*_E_ from 0.37 to 0.96. Significant heterozygote deficiency was observed for 2 markers (Esup6 and 9). Null alleles might occur at these loci, as confirmed by further analysis using the MICRO-CHECKER software [17]. We tested for linkage disequilibrium (LD) between all pairs of loci using the software GENETIX 4.05 [16]. The significance of the results was tested with 10000 permutations at the 5% level, and one pair showed significant LD (Esup3-Esup6, p = 0.09).

The high variability displayed by these microsatellite loci should be useful for assessing the genetic structure of *E. superba* at different spatial and temporal scales. In particular these novel markers should allow testing controversial hypotheses of spatial genetic structure versus panmixia in Antarctic krill, seasonal and inter-annual population variability, and effects of fishing pressure on population genetic diversity.

### Availability of supporting data

The microsatellite sequences are available through the National Centre for Biotechnology Information (see http://www.ncbi.nlm.nih.gov/). The accession numbers on the repository are the following: GenBank accession no. KF648623 through KF648632.

## Competing interests

The authors declare they have no competing interests.

## Authors’ contributions

GAP was responsible for the design and implementation of the study, supervision of the work and processing interpretation of the results. RC and ST participated in data analysis, microsatellite marker validation and drafted the manuscript. CMD coordinated the field sampling and participated in data interpretation. All authors read and approved the final manuscript.
